# Production of 17 strains of edible mushroom grown on corn stover and its effect on the chemical composition and ruminal *in vitro* digestibility of the residual substrate

**DOI:** 10.1371/journal.pone.0286514

**Published:** 2023-05-31

**Authors:** Angélica Valeria Lorenzana-Moreno, Hermilo Leal Lara, Luis Corona, Omar Granados, Claudia C. Márquez-Mota

**Affiliations:** 1 Departamento de Nutrición Animal y Bioquímica, Facultad de Medicina Veterinaria y Zootecnia, Universidad Nacional Autónoma de México, México City, México; 2 Departamento de Alimentos y Biotecnología, Facultad de Química, Universidad Nacional Autónoma de México, México City, México; 3 Departamento de Fisiología de la Nutrición, Instituto Nacional de Ciencias Médicas y Nutrición Salvador Zubirán, Ciudad de México, México; USDA Forest Service, UNITED STATES

## Abstract

The objective of this study was to evaluate the production (P) (g of fresh mushrooms /bag) and biological efficiency (BE) (g of fresh mushrooms per 100 g of dry substrate) of 17 fungal strains, namely *Pleurotus ostreatus* Po-IAP, Po-P38, Po-P14, Po-IE202, Po-Sfco, Po-JP, Po-Psma, and Po-POS, *Pleurotus djamour* Pd-PRO and Pd-UTMR, *Pleurotus eryngii* Pe-MB and Pe-PQ, *Lentinula edodes* L15, L9, L5, and LC, and *Hericium erinaceus* Heri, produced in corn stover (CS) and to assess the content of crude protein (CP), lignin (L), cellulose, hemicellulose, acid detergent fiber (ADF), and neutral detergent fiber (NDF) and *in vitro* digestibility of dry matter (IVDMD) of the residual substrate of CS, the so called spent mushroom substrate (SMS), in comparison to the non-inoculated substrate (C). The variables were analyzed as a completely randomized block design using R 4.0.3 software. Means were compared using Tukey’s procedure. The *Pleurotus* spp. strains, compared to *Lentinula* spp. and *Hericium* spp., presented better BE and P. In comparison to C, the SMS increased the CP content (p < 0.05) from 10.8% (Po-JP) to 70.3% (LC), while NDF decreased (p < 0.05) from 11.5% (Pd-Pro) to 33.5% (L15) and IVDMD increased (p < 0.05) from 16.2% (Heri) to 47.7% (Pd-UTMR). In conclusion, of the 17 strains evaluated, the 3 strains of *Lentinula edodes* (L5, L15, and L9), one strain of *Pleurotus djamour* (Pd-UTMR), and one strain of *Pleurotus ostreatus* (Po-IAP) generated a SMS that, due to its nutritional improvement and increase in IVDMD, could be used as feed for ruminants. Our results also showed that corn stover is a suitable substrate to produce *Pleurotus* spp. fruiting bodies, with strain Po-IAP as the best yielding.

## Introduction

The residue obtained from corn production is known as corn stover, and it plays a significant role in animal nutrition, especially in mixed systems where agricultural and livestock activities are combined. Corn stover has a low protein content (4.9%), low digestibility, elevated levels of highly lignified structural carbohydrates (64.54%), and low energy, mineral, and vitamin value [1]. Thus, different strategies are used to improve the nutritional quality of corn stover; the most used methods are physical, chemical, and biological treatments. High-pressure heat treatment is a physical method that breaks the lignocellulosic structures of agricultural residues; however, a disadvantage of this method is that due to the temperature and high pressure of the water, there is a probability that the cellulose present is hydrolyzed [2]. Chemical treatments have proven to be efficient in increasing digestibility; however, during these treatments, hazardous waste is generated in the environment [3].

Among biological processes, the cultivation of edible white rot fungi has proven to be a good strategy [4]. White rot fungi can grow on highly lignified residues, such as wheat straw, corn stover, sawdust, cotton husk, sorghum, oats, and coffee residues [5, 6], due to their capacity to hydrolyze lignin through the activity of extracellular ligninolytic enzymes [7]. The production of white rot fungi in agricultural wastes is an economic activity that has gained importance in recent years. It has been estimated that by 2026, the worldwide production of edible mushrooms will be 20.84 million tons [8]. To produce 1 kg of fresh mushroom, approximately 5 kg of residues, called spent mushroom substrate (SMS), are generated [9]. The SMS contains residues of fungal mycelia, modified or degraded lignin and ligninolytic enzymes, polysaccharides, vitamins, and trace elements, such as iron, calcium, zinc, and magnesium, which increase the nutritional value of this residue, allowing its use as an animal feedstuff [10, 11].

Previous studies have demonstrated that cultivation of *P*. *ostreatus* in corn straw substrate generates an SMS with increased CP content and reduced NDF content [12, 13], which improves the dry matter intake and average daily gain of Pelibuey lambs [12]. Tuyen [14] evaluated the capacity of 11 fungal strains to degrade lignin and its possible use as a ruminant feedstuff. Their results showed that only 3 strains: *Ceriporiopsis subermispora* (Pilat) Gilb. & Ryvarden 1985, *Lentinula edodes* (Berkeley) Pegler 1975, and *Pleurotus eryngii* var. *eryngii* Quél 1872, produced an increased *in vitro* degradability of wheat straw residual substrate and an elevated lignin–cellulose ratio [14]. Wang [11] evaluated the effect of the cultivation of four *Pleurotus* spp. (*Pleurotus djamour*, *Pleurotus eryngii*, *Pleurotus sajor-caju*, and *Pleurotus citrinopilautus*) on the nutritional value of corn stover and observed that the treatment with *Pleurotus sajor-caju* and *Pleurotus eryngii* caused a decrease in ADF and a significant increase in the essential amino acid content [11], indicating that the nutritional value and quality of the SMS depends on the type of substrate, fungal strain and genus [15, 16].

We hypothesized that depending on the cultivated fungal strain, the production (P) (g of fresh mushrooms /bag), biological efficiency (BE) (g of fresh mushrooms per 100 g of dry substrate), and nutritional quality (CP, NDF, ADF, and amino profile) of the SMS would differ. Thus, in the present study we evaluated the effect of different strains and genera of white rot fungi, namely *Pleurotus ostreatus* Po-IAP, Po-P38, Po-P14, Po-IE202, Po-Sfco, Po-JP, Po-Psma, and Po-POS, *Pleurotus djamour* Pd-PRO and Pd-UTMR, *Pleurotus eryngii* Pe-MB and Pe-PQ, *Lentinula edodes* L15, L9, L5, and LC, and *Hericium erinaceus* Heri, on the BE, nutritional value, and *in vitro* digestibility of corn stover SMS.

## Material and methods

### Animal management and treatments

The research protocol (MC-2018/2-24) was approved by the Institutional Committee for the Care and Use of Experimental Animals (SICUAE) of the Faculty of Veterinary Medicine and Zootechnics, National Autonomous University of Mexico (FMVZ, UNAM). Experiments were carried out at the Center for Practical Teaching and Research in Animal Production and Health (CEPIPSA). Three rumen fistulated male ovine (55 ± 0.5 kg) were used as ruminal fluid donors. The treatments were set up in a completely randomized block design.

### Fungal strains and cultivation

A total of 17 strains of white rot fungi were used in the experiment: *Pleurotus ostreatus*: Po-IAP, Po-P38, Po-P14, Po-IE202, Po-Sfco, Po-JP, Po-Psma, and Po-POS; *Pleurotus djamour*: Pd-PRO and Pd-UTMR; *Pleurotus eryngii*: Pe-MB and Pe-PQ; *Lentinula edodes*: L15, L9, L5, and LC; and *Hericium erinaceus*: Heri. All strains used in this study were stored in the culture collection of the laboratory of fungal biotechnology of the Faculty of Chemistry (UNAM). Substrate preparation and inoculation were performed using a commercial grower (Hongos el Encinal, Estado de México, Mexico). Wheat grain was used to prepare spawn of the different fungal strains. The wheat grain was washed and cooked in boiling water for 45 minutes. Thereafter, excess water was drained, and the grain was washed with cold water. The grain was then supplemented with CaCO_3_ (1.3%) and CaSO_4_ (0.3%). Polypropylene autoclavable bags were filled with 500 g of cooked wheat grain (3 replicates per mushroom strain) and autoclaved at 121°C and 15 lb/in^2^ for 2 hours. After sterilization, the grain was inoculated with mushroom mycelia and incubated for 15–20 days at 25°C.

The substrate for mushroom fructification was prepared with corn stover (80.55%), ground sorghum (5.89%), bran (3.89%), gluten (4.89%), CaCO_3_ (3.89%), and CaSO_4_ (0.89%). The dried materials were mixed, and water was added to obtain a humidity of 67%. Polypropylene autoclavable bags were filled with 3.5 kg of the substrate (3 replicates per mushroom strain) and autoclaved at 121°C and 15 lb/in^2^ for 3 hours. Bags with autoclaved substrate were allowed to cool at room temperature for 24 hours and then inoculated with 5% grain spawn. After incubation for 25 days at 24°C, the full-grown substrate was transferred to the Department of Food Science and Biotechnology, Faculty of Chemistry (UNAM) and placed in the fruiting room (19°C with 75–85% relative humidity and continuous ventilation with fresh air). The full-grown substrates were liberated from the upper part of the bag and placed on racks at a spacing of 15–20 cm. Fruiting bodies were cropped from substrates in an early stage of maturation, i.e., when the pileus cap was beginning to open. The weight of fresh mushrooms and number of mushrooms produced from each replicate were registered to calculate the production (P) (g of fresh mushrooms /bag) and BE (g of fresh mushrooms per 100 g of dry substrate) of each strain. After mushroom production, the residual substrate, i.e., SMS, was transported to the Department of Animal Nutrition and Biochemistry of the Faculty of Veterinary Medicine and Animal Science, UNAM for analysis.

### Chemical analysis

The chemical composition of the SMS obtained from each fungal strain was determined using protocols from the AOAC [17]. Neutral detergent fiber (NDF), acid detergent fiber (ADF), lignin, cellulose, and hemicellulose contents were also analyzed [18]. The protein fractions were determined in the SMS with a higher IVDMD, according to [19]. The mineral content in the SMS with a higher IVDMD was determined according to protocol 968.08 from the AOAC [17].

### Substrate analyses

The consumption of substrate, lignin, cellulose, hemicellulose, lipids, crude protein, ADF, and NDF consumption and the IVDMD increase were determined for each fungal strain. The calculations are shown in Eqs 1–3.


$$Substrate\;consumption=\frac{g\;Ash\;(SMS)-g\;Ash\;(control)}{g\;Ash\;(control)}\times 100$$ (1)

$$Component\;consumption=\frac{\frac{g\;Component\;(control)}{g\;Ash\;(control)}-\frac{g\;Componet\;(SMS)}{g\;Ash\;(SMS)}}{\frac{g\;Component\;(control)}{g\;Ash\;(control)}}\times 100$$ (2)

$$IVDMD\;increase=\frac{IVDMD\;(SMS)-IVD\;MD\;(control)}{IVDMD\;(control)}\times 100$$ (3)

### *In vitro* dry matter digestibility

The *in vitro* dry matter digestibility was estimated as described by Tilley and Terry [20]. Briefly, rumen fluid was collected through the rumen cannula from three male Pelibuey sheep (55 ± 1 kg, 1 year old) fed a basal diet consisting of corn stover, alfalfa hay, and corn silage. Rumen fluid was collected in the morning before the animals were fed, filtered to eight layers of cheesecloth, and preserved under anaerobic conditions at a temperature of 39°C. Subsequently, rumen fluid was mixed with reduced and mineral solutions [21] in a ratio of 1:9 v/v to obtain the rumen inoculum [22]. Previously, 250 mg dry matter (DM) of the 17 SMS and non-inoculated substrate, used as a control (ground in a Model 4 Wiley® mill, through a 1-mm screen), were placed in 50 mL conical tubes. Afterwards, 25 mL of rumen inoculum was added and capped with rubber stoppers. The tubes were placed in a water bath with lateral oscillation (30/min) at 39°C for 48 h. After this time, the tubes were centrifuged at 3000 rpm for 10 min. The supernatant was discarded, and the pellet was suspended in 25 mL of pepsin solution previously heated at 40°C. The tubes were capped with rubber stoppers and placed in a water bath with lateral oscillation (30/min) at 39°C for 48 h. After the incubation period, the content of the tube was filtered using a filter paper disc (Whatman No. 41), a Büchner funnel, and a vacuum pump. After filtering, the filter paper discs were placed in a forced-air oven at 55°C for 48 h and weighed (Ohaus-Explorer® model AX12478, México) to determine residual DM.

### Determination of amino acids

The concentration of amino acids in the SMS with a higher IVDMD was determined by HPLC (triplicate). A total of 400 mg of SMS was hydrolyzed with 4 mL of HCl 0.1 M and 150 μL of the hydrolyzed sample was mixed with 38 μL of trichloroacetic acid, incubated for 30 min at 4°C and centrifuged at 4°C at 13,500 rpm for 12 min. The supernatant was used for the derivatization of amino acids with OPA (o-Phthalaldehyde) and FMOC (9-fluorenylmethylchloroformate). A sample of 0.5 μl was injected into an analytical column, Agilent ZORBAX Eclipse AAA (size 4.6 × 150 mm, 5 μm) coupled to a fluorescence detector at 340 nm excitation and 450 nm emission (Agilent G1321B) for analysis. The HPLC Agilent 1260 Infinity system was coupled to a binary pump (Agilent G1312B) and a robotic auto-sampler (Agilent G1367B).

### Statistical analysis

The data from the chemical analysis were analyzed with the agricolae package in R 4.0.3 software. Values were compared by a one-way ANOVA with Tukey’s post-hoc test (p < 0.05), according to the following model:

$$Yij=\mu_{i}+\iota_{i}+\varepsilon_{ij}$$

where Yij = response variable, μ = general mean, ι_i_ = effect of SMS type at level i, j, and ε_ij_ = effect of random error.

The data from the *in vitro* dry matter digestibility assay were analyzed as a completely randomized block design with the agricolae package in R 4.0.3 software. Values were compared by a one-way ANOVA with Tukey’s post-hoc test (p < 0.05), according to the following model:

$$Y_{ijk}=\mu +\tau_{i}+\beta_{j}+\varepsilon_{ijk}$$

Where Y_ijk_ = response variable, μ = general mean, τ_i_ = effect of SMS type at level i, β_j_ = effect of block at level j, and ε_ijk_ = effect of random error.

## Results

All fungal strains used in the present study grew in corn stover substrate (Table 1), but mushroom production varied widely among the strains. Strains Po-P14, Po-IE202, Po-JP, Po-Psma, Pd-UTMR, Pe-PQ, L15, and L5 showed the maximal significant yield (MSY) just after one week of production, whereas strains L9, LC, and Heri had an MSY at two weeks of production. Strains Po-Sfco, Po-POS, and Pe-MB reached MSY after 3 weeks, while strains Po-IAP, Po-P38, and Pd-PRO required 4 weeks. Most fungal strains reached a production close to or above 500 g of fresh mushrooms/bag except for 4 strains, i.e., Po-POS, Pe-PQ, L9, L5, and LC. Accordingly, except for these 4 strains, a BE above 40% was produced by all other fungal strains, with Po-IAP as the highest yielding (76% BE).

**Table 1:** Mushroom production (g fresh mushrooms/bag) and biological efficiency (%) at the week of maximal significant yield of fungal strains cultivated in corn stover substrate.

Fungal Strain	Week of maximal significant yield	Mushrooms production (g fresh mushrooms /bag)	Biological efficiency (g fresh mushrooms /100 g DM)
Po-IAP	4	881±84^a^	76±7^a^
**Po-P38**	4	697±90^ab^	60±8^ab^
**Po-P14**	1	623±156^ab^	54±14^ab^
**Po-IE202**	1	582±545^ab^	50±47^abc^
**Po-Sfco**	3	524±19^abc^	45±2^abc^
**Po-JP**	1	488±72^abc^	42±6^abc^
**Po-Psma**	1	475±106^abc^	41±9^abc^
**Po-POS**	3	219±217^bc^	19±19^bc^
**Pd-PRO**	4	605±41^ab^	52.4±4^ab^
**Pd-UTMR**	1	445±135^abc^	39±12^abc^
**Pe-MB**	3	585±182^ab^	51±16^ab^
**Pe-PQ**	1	374±200^abc^	32±17^abc^
**L15**	1	509±183^abc^	44±16^abc^
**L9**	2	408±92^abc^	35±8^abc^
**L5**	1	312±159^bc^	27±14^abc^
**LC**	2	30.2±30.2^c^	2.6±2.6^c^
**Heri**	2	438±71^abc^	38±6^bc^

Po-IAP: *Pleurotus ostreatus* IAP, Po-P38: *Pleurotus ostreatus* P38, P14: *Pleurotus ostreatus* P14, Po-IE202: *Pleurotus ostreatus* IE202, Po-Sfco: *Pleurotus ostreatus* Sfco, Po-JP: *Pleurotus ostreatus* JP, Po-Psma: *Pleurotus ostreatus* Psma, Po- Po-POS: *Pleurotus ostreatus* POS, Pd-PRO: *Pleurotus djamour* PRODISET, Pd-UTMR: *Pleurotus djamour* UTMR, Pe-MB: *Pleurotus eryngii* MB, Pe-PQ: *Pleurotus eryngii* PQ, L15: *Lentinula edodes* L15, L9: *Lentinula edodes* L9, L5: *Lentinula edodes* L5, LC: *Lentinula edodes* LC, Heri: *Hericium* sp.

The values are the mean ± SEM, n = 3. Means in a column without a common letter differ from each other, p < 0.05. Differences were determined by one-way ANOVA; Tukey’s test was used as a post-hoc test.

Changes in the chemical composition of the degraded substrate (SMS) varied according to the way each fungal strain consumed the different components of the lignocellulosic material (Table 2). The lignin content increased with 11 of the fungal strains, from an initial value of 11.6 to the highest value of 16.9 g lignin /100 g SMS with strain L15. The cellulose content decreased with 11 of the fungal strains; strains L9, L5, and L15 showed the highest decrease, from an initial value of 29.2 to 16.6, 17.6, and 18.0 g cellulose /100 g SMS, respectively. A slight increase (9.24%) (p < 0.05) was observed with strain Po-POS. The hemicellulose content was reduced with all fungal strains (p < 0.05); strain L15 showed the highest reduction among all treatments, from an initial content of 41.9 to 2.6 g hemicellulose /100 g SMS. The lipid content increased (p < 0.05) only with strain L9, from an initial content of 5.4 to 7.3 g lipids/100 g SMS. Notably, 12 of the 17 fungal strains showed an increase in crude protein content from an initial value of 7.4 g/100 g SMS (p 0.05), ranging from 8.2 to 12.6 g/100 g SMS for strains Po-JP and LC, respectively. The ADF value in SMS increased (p < 0.05) with strains Po-Psma, Po-POS, Pe-MB, and Pe-PQ, from an initial value of 43.5 to 48.2 g ADF /100 g SMS for strain Pe-MB, while the ADF value decreased with strains Pd-PRO, L15, L9, L5, LC, and Heri (to the lowest value of 37.3 g ADF /100 g SMS with strain L9). The NDF value increased (p < 0.05) from an initial value of 63.4 to 70.1 and 75.3 g NDF /100 g SMS with strains Po-Psma and Pe-MB, respectively, while it decreased with 6 of the fungal strains (Pd-PRO, L15, L9, L5, LC, and Heri) to the lowest value of 42.1 g NDF /100 g SMS with strain L15. Finally, the IVDMD values increased (p < 0.05) for all 17 strains, from an initial value of 41.9 up to a maximum value of 61.9 g IVDMD /100 g SMS with strain Pd-UTMR.

**Table 2:** Chemical composition and *in vitro* dry matter digestibility of SMS and non-inoculated substrate (control).

SMS (strains)	Composition of degraded substrate (Spent Mushroom Substrate) (g/100g)									
	Dry matter	Ash	Lignin	Cellulose	Hemicellulose	Lipids	Crude protein	ADF	NDF	IVDMD
**Po-IAP**	96.3^cde^	20.0^c^	12.1^efg^	24.3^fg^	20.3^cd^	5.7^b^	6.9^h^	41.9^e^	62.1^de^	56.6^bcd^
**Po-P38**	97.1^abcde^	19.2^cd^	13.7^cd^	26.9^de^	16.6^de^	5.5^bc^	7.3^gh^	44.4^bc^	61.0^def^	51.8^efg^
**Po-P14**	97.8^ab^	19.6^cd^	13.3^de^	27.5^cde^	19.7^d^	3.7^de^	8.3^ef^	44.4^bc^	64.1^cd^	55.7^bcde^
**Po-IE202**	97.4^abcd^	21.2^b^	13.7^cd^	23.3^gh^	20.2^cd^	4.9^bcd^	8.7^def^	42.8^de^	63.0^cd^	54.6^bcde^
**Po-Sfco**	98.5^a^	19.1^cd^	12.9^de^	26.9^de^	20.2^cd^	5.3^bc^	8.0^fg^	43.3^cde^	63.5^cd^	55.1^bcde^
**Po-JP**	98.3^a^	18.7^de^	14.8^bc^	25.9^ef^	21.3^cd^	5.7^b^	8.2^ef^	44.0^bcd^	65.3^bcd^	48.9^fg^
**Po-Psma**	97.7^abc^	19.4^cd^	11.5^g^	28.3^cd^	24.7^bc^	4.6^bcd^	7.3^gh^	45.4^b^	70.1^b^	56.9^bcd^
**Po-POS**	97.2^abcde^	17.1^f^	13.1^de^	31.9^a^	19.7^d^	5.0^bc^	8.6^def^	47.5^a^	67.3^bc^	53.2^def^
**Pd-PRO**	97.9^ab^	22.7^a^	12.1^efg^	22.2^hi^	17.5^de^	5.5^bc^	9.2^d^	38.6^fgh^	56.1^fg^	53.9^cde^
**Pd-UTMR**	97.5^abcd^	17.5^ef^	11.4^g^	28.3^cd^	19.3^d^	4.9^bcd^	7.2^h^	43.9^bcd^	63.2^cd^	61.9^a^
**Pe-MB**	96.8^bcde^	15.8^g^	12.7^def^	30.9^ab^	27.1^b^	4.5^bcd^	8.4^ef^	48.2^a^	75.3^a^	54.8^bcde^
**Pe-PQ**	97.5^abcd^	16.8^fg^	12.2^efg^	30.3^ab^	20.2^cd^	4.4^cde^	8.4^ef^	47.6^a^	67.8^bc^	53.1^defg^
**L15**	96.9^bcde^	19.6^cd^	16.9^a^	18.0^k^	2.6^h^	4.2^cde^	11.2^bc^	39.5^fg^	42.1^h^	58.2^abc^
**L9**	96.2^de^	20.0^bc^	15.7^ab^	16.6^k^	8.0^fg^	7.3^a^	11.6^b^	37.3^h^	45.3^h^	57.0^bcd^
**L5**	97.6^abc^	20.0^bc^	15.3^b^	17.6^k^	7.4^gh^	5.2^bc^	10.7^c^	38.1^gh^	45.4^h^	58.9^ab^
**LC**	97.1^abcde^	17.8^ef^	15.3^b^	20.3^j^	12.7^ef^	5.1^bc^	12.6^a^	39.8^f^	52.5^g^	52.1^efg^
**Heri**	97.6^abc^	16.7^fg^	14.8^bc^	20.5^ij^	17.5^de^	3.2^e^	8.8^de^	39.7^f^	57.2^efg^	48.7^g^
**Control**	95.9^e^	12.4^h^	11.6^fg^	29.2^bc^	41.9^a^	5.4^bc^	7.4^gh^	43.5^cd^	63.4^cd^	41.9^h^
***P-value***	0.05	0.05	0.05	0.05	0.05	0.05	0.05	0.05	0.05	0.05
**SEM**	0.1	0.27	0.2	0.56	0.99	0.11	0.19	0.39	1.05	0.55

Control: non-inoculated substrate, Po-IAP: Pleurotus ostreatus IAP, Po-P38: Pleurotus ostreatus P38, P14: Pleurotus ostreatus P14, Po-IE202: Pleurotus ostreatus IE202, Po-Sfco: Pleurotus ostreatus Sfco, Po-JP: Pleurotus ostreatus JP, Po-Psma: Pleurotus ostreatus Psma, Po- Po-POS: Pleurotus ostreatus POS, Pd-PRO: Pleurotus djamour PRODISET, Pd-UTMR: Pleurotus djamour UTMR, Pe-MB: Pleurotus eryngii MB, Pe-PQ: Pleurotus eryngii PQ, L15: Lentinula edodes L15, L9: Lentinula edodes L9, L5: Lentinula edodes L5, LC: Lentinula edodes LC, Heri: Hericium sp ADF: acid detergent fiber, NDF: neutral detergent fiber, IVDMD: in vitro dry matter digestibility. The values are the mean, n = 6. SEM: standard error of the mean. Means in a column without a common letter differ from each other, p < 0.05. Differences were determined by one-way ANOVA; Tukey’s test was used as a post-hoc test.

Notably, Table 2 shows that ash content increased (p < 0.05) with all fungal strains, and *P*. *djamour* (Pd-PRO) showed the highest increase, from an initial value of 12.4 to 22.7 g ash /100 g SMS, corresponding to an 85.5% increase in ash content. The increase in ash content was directly related to the consumption of the substrate, as indicated in Table 3, with strains Pd-PRO and Po-IE302 producing the highest substrate consumption (45.3 and 41.5%, respectively). The consumption of substrate components shown in Table 3 was calculated relative to the changes in chemical composition of SMS with the changes in ash content of the corresponding SMS according to Eq (2). Thus, the highest (p < 0.05) consumption of lignin was observed with strain Pd-PRO (42.7%), while *L*. *edodes* strains L-9, L-5, and L-15 showed the highest cellulose consumption (64.8, 62.7, and 61%), and all *L*. *edodes* strains (L-15, L-5. L-9 and LC) had the highest hemicellulose consumption (96.1, 89.1, 88.2, and 78.9%, respectively). The combined consumption of lignin and polysaccharides was reflected in the consumption of ADF and NDF; thus, *P*. *djamour* strain Pd-PRO registered the highest (p < 0.05) consumption of ADF (51.4%), and *L*. *edodes* strain L-15 had the highest consumption of NDF (58.1%). Following the same rationale, the highest (p < 0.05) consumption of lipids (56.3%) was produced by *P*. *ostreatus* strain Po-P14, while strain Po-IAP produced the highest consumption of crude protein (42.3%). These statements, however, have to be considered cautiously, since the initial lipid and crude protein values in the control substrate were basically provided by 3 ingredients, i.e., sorghum (5.89%), bran (3.89%), and gluten (4.89%), and as mycelium grows, the ash content decreases in essence due to consumption of the lignocellulose complex but at the same time crude protein is produced by the fungal biomass, although probably with a lower rate and reflected as a consumption of crude protein. The highest increase (p < 0.05) in IVDMD (47.8%) was registered with *P*. *djamour* strain Pd-UTMR. The increase in IVDMD in SMS is a result of complex changes in substrate composition occurring due to fungal degradation of lignocellulose, and it is interrelated with the overall consumption of substrate components and the production of fungal biomass. Analysis of the data presented in Table 3 indicate a positive relationship between the increase in IVDMD and the consumption of lignin (r = 0.2987, p = 0.05), hemicellulose (r = 02375, p = 0.0515), NDF (r = 0.231, p = 0.057), and substrate consumption (r = 0.223¸ p = 0.067).

**Table 3:** Consumption of substrate, substrate components, and increase of *in vitro* dry matter digestibility.

SMS (strains)	Consumption of substrate (g/100 initial substrate)	Consumption of substrate component (g of component consumed / g ash)							IVDMD Increase (%)
		Lignin	Cellulose	Hemicellulose	Lipids	Crude protein	ADF	NDF	
**Po-IAP**	37.9^bc^	35.1^abc^	48.3^d^	69.9^cdef^	34.8^bcd^	42.3^a^	40.2^cd^	39.2^cd^	35.2^bcde^
**Po-P38**	35.3^cd^	23.6^def^	40.5^e^	74.4^bcd^	34.2^bcd^	36.9^ab^	33.9^e^	37.8^cde^	23.7^fg^
**Po-P14**	36.7^cd^	27.4^cde^	40.4^e^	70.2^cdef^	56.3^a^	29.7^bc^	35.4^de^	36.1^cde^	32.3^bcdef^
**Po-IE202**	41.5^ab^	31.1^bcd^	53.5^cd^	71.8^bcde^	47.2^abc^	31.5^bc^	42.5^bc^	42.0^c^	30.5^bcdef^
**Po-Sfco**	35.1^cd^	27.4^cde^	40.3^e^	68.8^def^	35.4^bcd^	30.4^bc^	35.3^de^	35.1^def^	31.5^bcdef^
**Po-JP**	33.4^de^	15.0^ghi^	40.9^e^	66.1^ef^	29.6^de^	26.5^c^	32.6^ef^	31.5^ef^	16.9^g^
**Po-Psma**	36.1^cd^	36.9^ab^	38.1^e^	62.3^f^	45.0^abcd^	37.6^ab^	33.4^ef^	29.5^fg^	36.0^bcd^
**Po-POS**	27.6^fg^	18.5^fgh^	20.9^gh^	65.9^ef^	32.3^cde^	15.7^d^	20.8^g^	23.2^gh^	27.1^defg^
**Pd-PRO**	45.3^a^	42.7^a^	58.4^bc^	77.1^bc^	44.3^abcd^	32.6^bc^	51.4^a^	51.7^b^	28.9^cdef^
**Pd-UTMR**	29.1^efg^	30.5^bcde^	31.4^f^	67.4^def^	35.4^bcd^	31.2^bc^	28.4^f^	29.5^fg^	47.8^a^
**Pe-MB**	21.4^h^	13.8^hi^	16.9^h^	49.2^g^	33.8^bcd^	11.7^de^	12.9^h^	6.8^i^	31.0^bcdef^
**Pe-PQ**	26.1^fg^	22.4^efg^	23.4^g^	64.4^ef^	39.8^abcd^	16.4^d^	19.0^g^	21.0^h^	26.8^defg^
**L15**	38.0^bc^	7.8^ij^	61.0^ab^	96.1^a^	50.1^ab^	4.4^e^	42.5^bc^	58.1^a^	39.2^abc^
**L9**	38.0^bc^	15.6^fghi^	64.8^a^	88.2^a^	16.3^e^	3.7^e^	48.8^ab^	55.8^ab^	36.2^bcd^
**L5**	36.7^cd^	18.1^fgh^	62.7^ab^	89.1^a^	40.2^abcd^	10.9^de^	45.7^b^	55.6^ab^	40.6^ab^
**LC**	30.3^ef^	8.2^ij^	51.6^d^	78.9^b^	34.0^bcd^	-17.8^f^	36.3^de^	42.4^c^	24.4^efg^
**Heri**	25.7^gh^	4.9^j^	47.9^d^	69.0^cdef^	55.3^a^	11.7^de^	32.2^ef^	33.1^def^	16.3^g^
***P-value***	0.05	0.05	0.05	0.05	0.05	0.05	0.05	0.05	0.05
**SEM**	0.761	1.34	1.74	1.37	1.37	1.89	1.23	1.63	1.06

Control: non-inoculated substrate, Po-IAP: *Pleurotus ostreatus* IAP, Po-P38: *Pleurotus ostreatus* P38, P14: *Pleurotus ostreatus* P14, Po-IE202: *Pleurotus ostreatus* IE202, Po-Sfco: *Pleurotus ostreatus* Sfco, Po-JP: *Pleurotus ostreatus* JP, Po-Psma: *Pleurotus ostreatus* Psma, Po- Po-POS: *Pleurotus ostreatus* POS, Pd-PRO: *Pleurotus djamour* PRODISET, Pd-UTMR: *Pleurotus djamour* UTMR, Pe-MB: *Pleurotus eryngii* MB, Pe-PQ: *Pleurotus eryngii* PQ, L15: *Lentinula edodes* L15, L9: *Lentinula edodes* L9, L5: *Lentinula edodes* L5, LC: *Lentinula edodes* LC, Heri: *Hericium* sp ADF: acid detergent fiber, NDF: neutral detergent fiber, IVDMD: *in vitro* dry matter digestibility. The values are the mean, n = 6. SEM: standard error of the mean. Means in a column without a common letter differ from each other, p < 0.05. Differences were determined by one-way ANOVA; Tukey’s test was used as a post-hoc test.

Since the SMS produced by strains Pd-UTMR, L5, and L15 showed the highest increases of IVDMD (47.8, 40.6, and 39.2%, respectively), their mineral profile, protein fractions (Tables 4 and 5), and amino acid content (Table 6) were compared to the control non-inoculated substrate. As shown in Table 4, the SMS produced with these 3 strains showed a significant (p < 0.05) increase in the concentration of iron (2.3 to 2.9-fold), calcium (1.5 to 2.3-fold), manganese (1.8-fold), magnesium (1.6-fold), and potassium (1.3-fold) in comparison to the non-inoculated substrate. Likewise, the sodium concentration was 2.7- and 1.6-fold higher (p < 0.05) in the SMS from L5 and Pd-UTMR, while zinc levels were 1.4- and 1.2-fold higher (p < 0.05) in the SMS from L15 and L5 than in the control non-inoculated substrate.

**Table 4:** Mineral profile of SMS with higher *in vitro* dry matter digestibility and non-inoculated substrate (control).

Mineral profile	SMS (Strains)				SEM	Changes (Proportion vs control substrate)		
						SMS (Strains)		
	Pd-UTMR	L5	L15	Control		Pd-UTMR	L5	L15
**Fe (mg/100g)**	124.21c	161.77a	134.78b	54.89d	10.9	2.3	2.9	2.5
**Ca (g/100g)**	5.68^a^	3.63^c^	4.11^b^	2.44^d^	0.38	2.3	1.5	1.7
**Mn (mg/100g)**	10.82^a^	10.58^a^	9.72^b^	5.89^c^	0.57	1.8	1.8	1.7
**Mg (mg/100g)**	299.78^a^	308.92^a^	301.94^a^	186.21^b^	13.69	1.6	1.7	1.6
**K (g/100g)**	1.77^a^	1.71^b^	1.80^a^	1.40^c^	0.05	1.3	1.2	1.3
**Na (mg/100g)**	98.73^b^	167.33^a^	64.48^c^	62.18^c^	13.29	1.6	2.7	1
**Zn (mg/100 g)**	2.41^d^	3.80^b^	4.72^a^	3.26^c^	0.22	0.7	1.2	1.4

SMS: spent mushroom substrate; fungal strains: *Pleurotus djamour* Pd-UTMR, *Lentinula edodes*: L5, L15; Control: non-inoculated substrate. The values are the mean, n = 4. SEM: standard error of the mean. Means in a row without a common letter differ from each other, p < 0.05. Differences were determined by one-way ANOVA; Tukey’s test was used as a post-hoc test.

**Table 5:** Protein fraction of SMS with higher *in vitro* dry matter digestibility and non-inoculated substrate.

Protein fraction (%)	SMS (Strains)				SEM	Changes (Proportion vs control substrate)		
						SMS (Strains)		
	Pd-UTMR	L5	L15	Control		Pd-UTMR	L5	L15
**A**	1.93^a^	1.87^a^	2.33^a^	1.19^b^	0.14	1.6	1.6	2
**B1**	1.97^a^	1.66^a^	1.79^a^	0.96^b^	0.12	2.1	1.7	1.9
**B2**	3.28^bc^	3.35^b^	4.4^a^	2.94^c^	0.17	1.1	1.1	1.5
**B3**	0.53^c^	2.44^a^	0.7^c^	1.49^b^	0.23	0.4	1.6	0.5
**C**	0.17^d^	1.23^b^	2.45^a^	0.96^c^	0.25	0.2	1.3	2.6

SMS: spent mushroom substrate; fungal strains: *Pleurotus djamour* Pd-UTMR, *Lentinula edodes*: L5, L15; Control: non-inoculated substrate. The values are the mean, n = 4. SEM: standard error of the mean. Means in a row without a common letter differ from each other, p < 0.05. Differences were determined by one-way ANOVA; Tukey’s test was used as a post-hoc test.

**Table 6:** Amino acid content of SMS with higher *in vitro* dry matter digestibility and non-inoculated substrate (control).

Amino acids (mM)	SMS (Strains)				Changes (Proportion vs control substrate)		
					SMS (Strains)		
	Pd-UTMR	L5	L15	Control	Pd-UTMR	L5	L15
**Met**	2.6 ± 0.01^c^	23.7 ± 3.3^a^	14.1 ± 0.3^b^	0.0 ± 0.0^d^	26	237	141
**Asn**	20.9 ± 1.4^a^	6.1 ± 0.6^b^	5.4 ± 0.04^b^	3.0 ± 0.09^b^	7.0	2.0	1.8
**Ser**	51.9 ± 1.5^a^	40.7 ± 2.1^b^	14.9 ± 0.6^c^	15.4 ± 0.2^c^	3.4	2.6	1.0
**Lys**	42.0 ± 1.1^a^	31.4 ± 4.4^a^	17.5 ± 2.0^b^	14 ± 1.2^b^	3.0	2.2	1.3
**Thr**	127.5 ± 12.1^a^	108.7 ± 7.7^ab^	82.4 ± 3.9^bc^	58.7 ± 0.2^c^	2.2	1.9	1.4
**Glu**	183.3 ± 12.2^a^	109.1 ± 10.1^b^	149.3 ± 0.3^a^	87.6 ± 0.7^b^	2.1	1.2	1.7
**Gln**	118.5 ± 6.8^a^	63.3 ± 2.5^b^	57.8 ± 0.05^b^	66.3 ± 0.7^b^	1.8	1.0	0.9
**Leu**	155.6 ± 0.7^a^	100.5 ± 4.2^b^	92.3 ± 0.5^bc^	87.7 ± 0.7^c^	1.8	1.1	1.1
**His**	14.9 ± 1.0^a^	14.6 ± 1.1^a^	13.9 ± 0.6^a^	8.4 ± 0.05^b^	1.8	1.7	1.7
**Phe**	41.9 ± 5.3^a^	36.4 ± 2.4^ab^	28.5 ± 0.2^b^	28.6 ± 0.2^b^	1.5	1.3	1.0
**Gly**	92.7 ± 3.7^a^	37.7 ± 0.9^c^	41.5 ± 0.3^c^	67.8 ± 1.4^b^	1.4	0.6	0.6
**Ile**	79.3 ± 0.2^a^	48.4 ± 1.5^c^	57.9 ± 0.1^b^	60.1 ± 0.4^b^	1.3	0.8	1.0
**Val**	117.1 ± 3.3^a^	70.4 ± 1.9^d^	82.9 ± 0.4^c^	95.8 ± 0.4^b^	1.2	0.7	0.9
**Trp**	2.8 ± 0.17^b^	3.7 ± 0.2^b^	6.4 ± 0.02^a^	3.3 ± 0.4^b^	0.8	1.1	1.9
**Ala**	245.7 ± 22.2^b^	184.2 ± 8.7^c^	283.5 ± 5.3^b^	382.9 ± 4.7^a^	0.6	0.5	0.7
**Tyr**	41.9 ± 4.7^c^	39.1 ± 2.9^c^	66.2 ± 8.7^b^	89.9 ± 0.7^a^	0.5	0.4	0.7
**Asp**	110 ± 0.8^b^	76.9 ± 0.6^c^	37.4 ± 0.8^d^	300.4 ± 3.6^a^	0.4	0.3	0.1
**Pro**	131.7 ± 5.2^b^	89.3 ± 1.6^c^	115.6 ± 9.9^bc^	372.8 ± 8.7^a^	0.4	0.2	0.3
**Arg**	20.4 ± 0.2^c^	103.1 ± 7.2^a^	71.1 ± 5.0^b^	73.3 ± 1.4^b^	0.3	1.4	1.0

SMS: spent mushroom substrate; fungal strains: Pleurotus djamour Pd-UTMR, Lentinula edodes: L5, L15; Control: non-inoculated substrate The values are the mean, n = 3. SEM: standard error of the mean. Means in a row without a common letter differ from each other, p < 0.05. Differences were determined by one-way ANOVA; Tukey’s test was used as a post-hoc test.

Regarding the values of the protein fraction in SMS (Table 5), the 3 strains produced increases (p < 0.05) in fraction A (1.6- to 2-fold), fraction B1 (1.7- to 2.1-fold) and fraction B2 (1.1- to 1.5-fold). With fraction B3, a 1.6-fold increase (p < 0.05) was observed with strain L5, while it decreased 50% with strains Pd-UTMR and L15. Concerning protein fraction C, it increased (p < 0.05) in SMS from L15 and L5 (2.6- and 1.3-fold, respectively), whereas it decreased to 20% with strain Pd-UTMR.

The amino acid content of the SMS from these strains is shown in Table 6. Remarkably, an increase (p < 0.05*)* in the concentration of 13, 8, and 5 amino acids was observed in the SMS from strains Pd-UTMR, L5, and L15, respectively. Notably, methionine was absent in the control non-inoculated substrate, but it was bio-synthesized by all 3 strains (L5, L15, and Pd-UTMR), reaching final concentrations in the SMS of 23.7, 14.1, and 2.6 mM, respectively. Similarly, the concentrations of threonine, glutamic acid, and histidine were also increased (p < 0.05*)* with the 3 fungal strains. Strain Pd-UTMR increased (p < 0.05*)* the concentrations of asparagine (7-fold), serine (3.4-fold), lysine (3-fold), glutamine (1.8-fold), and leucine (1.8-fold), while strain L5 only increased (p < 0.05*)* the concentrations of serine (2.6-fold), lysine (2.2-fold), and leucine (1.1-fold).

## Discussion

Our results show that corn stover is a suitable substrate to produce edible mushrooms, and that the SMS from Pd-UTMR, L5, and L15 showed increased IVDMD in comparison to the SMS of the different strains evaluated in the present study, indicating that this residue might be valuable for animal nutrition.

The production of edible fungi on highly lignified residues is a well-established process. Although corn stover is a lignocellulosic material, it is not normally used as a major ingredient of substrates to produce edible fungi because the yields obtained are lower than when mixtures of straw with nutrient-rich supplements are used. Since corn stover is the main agricultural residue in Mexico, mushroom production on such substrates in rural areas might be advantageous. Such a process may well have a double impact if, in addition to the utilization of corn stover accumulated in the fields, the SMS would be suitable for feeding ruminants.

The BE (g of fresh mushrooms per 100 g of dry substrate) is a measure of the efficiency of the conversion of substrate into mushroom fruiting bodies; high values indicate optimal use of the substrate, and low levels are associated with a defective assimilation of nutrients in the substrate by fungi [23]. In our study, we observed BE values in the range of 30–70%; therefore, most of the fungal strains used in the present study could be grown in corn stover substrate. The 2 strains with a BE over 60% (Po-IAP and Po-P38) are good candidates for commercial production. In addition to the yields of mushrooms, the viability of mushroom production is also associated with the timing at which the MSY is achieved [24]. As Table 1 shows, most fungal strains reached their MSY after 1 or 2 weeks of production, although the higher yielding strains, i.e., Po-IAP and Po-P38, required 4 weeks of production. Although further studies should be performed to define the viability of mushroom production with these strains, their behavior is encouraging.

Several studies have demonstrated that the cultivation of *Pleurotus* spp. fungi in rice straw [25], wheat straw [14], and corn stover [11, 26] enhances the nutritional value of SMS. As previously mentioned, SMS is a mix of residual fungal mycelium and modified lignocellulosic biomass with high levels of organic matter and extracellular ligninolytic enzymes [8, 11]. Although Niu [16] proposed that the composition of the SMS varies according to the cultivated fungal strain, no extensive evidence has been produced before our study with so many fungal species and strains [16]. The results shown in Table 2 indicate that cultivation of the different strains of *P*. *ostreatus*, *P*. *djamour*, *P*. *eryngii¸ L*. *edodes*, and *Hericium* spp. in substrates formulated with corn stover generated a residue (SMS) with increased nutritional value. Regarding its suitability as ruminant feed, a decrease in lignin content and increased amounts of hemicellulose and cellulose are of utmost importance. These parameters, as well as the IVDMD values, were followed in SMS from the various fungal strains. IVDMD increased in all cases, reaching a maximum value with strain Pd-UTMR (61.9 g IVDMD /100 g SMS).

Improving dry matter digestion in fibrous feeds used to feed ruminants is crucial, as insufficient fiber digestion reduces production profitability by limiting consumption and, consequently, animal productivity, which increases manure production. Different authors have reported that a one-unit increase in forage NDF digestibility is associated with an increase of 0.17 kg/d in dry matter intake (DMI) due to a more rapid hydrolysis of NDF, which could permit its faster disappearance from the rumen by a higher passage rate, as well as an increase of 0.25 kg/d in milk production due to the higher energy intake resulting from the increase in dry matter intake [27, 28].

Since enteric methane emissions are directly related to the amount and type of feed consumed, the increase in forage digestibility also has a direct effect on ruminant greenhouse gas emissions. Fermentation of fibrous feeds results in higher methane production and lower energy supply than concentrated feeds [29].

Table 2 also shows that the ash content increased significantly in all SMSs. This increase in ash content is a direct indication of substrate degradation; i.e., as organic materials are consumed, ash content increases. Although those fungal strains producing SMS with higher ash content are more adapted for growing in corn stover, differences in their capability to consume the different substrate components were clearly observed (Table 3). Interestingly, the data in Table 3 allowed us to establish for the first time that IVDMD increase is positively related to the consumption of lignin (r = 0.2987, p = 0.05), hemicellulose (r = 02375, p = 0.0515), NDF (r = 0.231, p = 0.057), and overall substrate consumption (r = 0.223¸ p = 0.067). Thus, overall consumption of the substrate and selective consumption of lignin over polysaccharides are crucial for obtaining an SMS that is most suitable as ruminant feed. The increased concentration of minerals in SMS, particularly Fe, Ca, Mg, Mn, and K, would improve the nutritional value of this material if these minerals are assimilated within the fungal biomass [30].

Minerals are essential for the proper functioning of a vast array of tissues and physiological processes. Ca, Mg, and P are essential macroelements for bone formation, muscle contractions, nerve transmission, energy metabolism, and blood clotting. It is necessary to ensure adequate consumption of these nutrients during certain productive stages of ruminants, such as the last third of gestation, lactation, and growth [31, 32]. Mineral deficiency can produce clinical symptoms of varying severity and is frequently associated with impaired immunity, stunted growth, reproductive disorders, and diminished productivity in animals [31, 33]. Mokolopi (2019) stated that grazing animals must be supplemented with P and Mg to prevent diseases such as pasture tetany [34]. Our findings permit us to hypothesize that SMS is an excellent source of the major mineral macroelements, which can reduce deficiencies of these elements in animals and, as a result, improve animal welfare.

It is not surprising that the lipid content of most SMS is comparable the control substrate, as mushrooms normally have a limited lipid content of 1.75–15.5% [35, 36]. The fruiting bodies of *Pleurotus* spp. and *Lentinula edodes* are considered good protein sources. Depending on the species, *Pleurotus* has a protein content ranging from 9.29 to 37.4% [37], while *Lentinula edodes* has a protein content of 14.87 to 27.13% [38]. Thus, the increase in crude protein content in the SMS observed in this study could be attributed to the fungal mycelium in the SMS. In 7 of the 17 treatments, a decrease in the ADF content was observed with a simultaneous increase in the lignin content and a decrease in the proportion of cellulose in the SMS of most of these treatments. Our results do not agree with studies that show that the treatment of highly lignified residues with white rot fungi lowers the lignin content [39], we observed that this pattern varied depending on the species and strains. White rot fungi, such as *Pleurotus* spp., produce a variety of lignocellulolytic enzymes that are able to degrade lignin and change its structure [40], allowing for the selective consumption of cellulose and hemicellulose polymers as an energy source [4]. Considering the lignin content of SMS, a decrease in the IVDMD was anticipated; however, our results showed that all the SMS showed higher digestibility (IVDMD) in comparison with the control. This could be attributed to the lignocellulolytic enzyme activity of the fungus, which generated fragments of lignin of low molecular weight, allowing fast fermenting carbohydrates to be more accessible to the rumen microorganisms.

However, our results showed that the cultivation of *Lentinula* and *Hericium* spp. strains produced a decrease in the content of NDF and hemicellulose. The NDF content is associated with DM consumption [41]; therefore, we can predict that animals fed with these residues would have a lower consumption of dry matter and, consequently, higher productivity. However, more studies are needed to confirm this hypothesis.

Another component that influences the digestibility of feedstuffs is protein content. Previous studies HAVE demonstrated that the protein fractions have an important role in the digestibility of feedstuffs [42, 43]. We observed an increase in protein fractions A, B1, and B2 in the SMS that had A higher IVDMD (Pd-UTMR, L5, and L15). Fraction A is the non-protein nitrogen [42]; this fraction is used by the microorganisms of the rumen for the synthesis of microbial proteins that pass to the intestine, where they are digested and contribute to the amount of metabolizable protein of the ruminant [44]. Fraction B1 is an easily degradable protein [42]; elevated values of fractions A and B1 are desirable in feedstuffs since they are rapidly degraded in the rumen, resulting in greater animal performance [45]. Fraction B2 is an intermediately degradable protein [42], it has been reported that 70–85% of this fraction is used in the rumen, and the remaining protein fraction is completed digested in the abomasum and small intestine [46].

These three fractions correspond mainly to rumen degradable protein, which is necessary for rumen microorganisms to achieve maximum carbohydrate digestion, as well as maximum microbial protein synthesis. A low level of rumen-degradable protein can decrease rumen NH_3_-N levels, dry matter intake, and microbial protein. Several authors [47, 48] mentioned that increasing the levels of degradable protein in the rumen significantly favors the digestibility of the nutrients, the fermentation of the rumen, and the synthesis of microbial proteins, which coincides with what was reported in this study. The increase in non-degradable protein in the rumen and the subsequent increase in digestibility indicate that SMS can be used in ruminant feeding. More studies are needed to evaluate the effect of SMS on microbial protein synthesis.

Thus, the observed increase in this protein fraction could explain that even though we observed an increase in the lignin content and a decrease in the cellulose and hemicellulose content, there was a higher IVDMD in the SMS from Pd-UTMR, L5, and L15. Protein fraction C represents the proteins associated with lignin, tannin, and Maillard products and is therefore highly resistant to ruminal microbial activity and is not a source of amino acids to ruminants [42]. Although we observed an increase in this fraction in the SMS from L5 and L15, the augmented content of the protein fractions did not affect the IVDMD.

Our results indicate that the amino acid profile of SMS depends on the cultivated fungal strain. A significant increase in the content of five limiting amino acids for ruminants (Met, Lys, Thr, Leu, and His) was observed in the SMS from Pd-UTMR, L5, and L15 compared with the non-inoculated substrate. Giallongo [49] reported that supplementation of a diet deficient in metabolizable protein with protected Lys increased the protein concentration in milk from 3.0 to 3.13% compared to the group without supplementation. The addition of protected His increased the DM intake and increased the milk protein content from 3.0 to 3.11%, and the combination of protected Met, Lys, and His further increased milk fat and protein yields. In addition, despite the fact that amino acids are primarily used as protein building blocks, they are also involved in a large number of metabolic functions [32]; thus, not only will they alter the productive parameters of animals, but they can also affect their health processes. In this regard, Khan [50] reported that the addition of Lys and Met to the diet of dairy cattle, particularly during the peripartum period, increases the antioxidant capacity, resulting in the regulation of immunity and anti-inflammatory status in animals.

Lastly, it should be mentioned that the improvement in the nutritional value of the SMS is not solely determined by the increase in the amount of limiting amino acids. Recent studies indicate that ruminal microorganisms of adult steers and sheep also have a limited capacity to metabolize extracellular L-glutamate due to little or no cell absorption [51]. Thus, there is growing interest in the effects of supplementing this in ruminants. According to Padunglerk [52], dietary supplementation with 5, 10, and 15% monosodium glutamate by-product (providing 0.24, 0.48, and 0.72% glutamate supplementation in the diet) increased milk production gain by 15, 22, and 33%, respectively. In the present study, a significant increase in Glu was also observed, particularly in the Pd-UTMR SMS, compared to the non-inoculated substrate (118.5 vs. 66.3 mM, respectively). More studies are needed to understand the possible relationship between the amino acid profile of SMS and its potential effect on rumen fermentation.

## Conclusions

Of the 17 fungal strains analyzed in the present study, two strains of *Lentinula edodes* (L5 and L15) and one strain of *Pleurotus djamour* (Pd-UTMR) generated a residue that, due to its nutritional enhancement and increased IVDMD, could be used as ruminant feed. Our results demonstrated that corn stover is a suitable substrate for producing *Pleurotus* spp. Further studies are recommended to elucidate the enzymatic mechanism by which these fungal strains hydrolyze lignocellulolytic residues in corn stover.

## Supporting information

S1 Table Mushroom production (g fresh mushroom /bag) and biological efficiency (g fresh mushroom/100 g DM). (XLSX)

S2 Table Chemical composition and in vitro dry matter digestibility of SMS and non-inoculated substrate (control). (XLSX)

S3 Table Mineral profile of SMS with higher in vitro dry matter digestibility and non-inoculated substrate (control). (XLSX)

S4 Table Protein fraction of SMS with higher in vitro dry matter digestibility and non-inoculated substrate (control). (XLSX)

S5 Table Amino acid content of SMS with higher in vitro dry matter digestibility and non-inoculated substrate (control). (XLSX)
